# Play Nicely Program in the prevention of violence against children: strengthening sustainable development^*^

**DOI:** 10.1590/1518-8345.7320.4434

**Published:** 2025-01-27

**Authors:** Janaina Recanello Begui, Andressa Larissa Dias Müller de Souza, Naiara Barros Polita, Maria de Fátima Garcia Lopes Merino, Adriana Valongo Zani, Rosângela Aparecida Pimenta

**Affiliations:** 1 Universidade Estadual do Norte do Paraná, Departamento de Enfermagem, Bandeirantes, PR, Brazil.; 2 Universidade Estadual de Londrina, Londrina, PR, Brazil.; 3 Universidade Estadual de Londrina, Departamento de Enfermagem, Londrina, PR, Brazil.; 4 Universidade Estadual de Maringá, Departamento de Enfermagem, Maringá, PR, Brazil.

**Keywords:** Child Welfare, Child Abuse, Parenting, Child Rearing, Primary Health Care, Sustainable Development

## Abstract

**Objective:**

to understand the perception of teachers and health professionals regarding the use of the Play Nicely Program for parents/caregivers in the prevention of violence against children.

**Method:**

a descriptive and exploratory qualitative study was conducted through three focus groups with twenty primary school teachers and primary health care professionals who implemented the Program for parents/caregivers in 2022. The data analysis was guided by French discourse analysis, interpreted through the lens of Urie Bronfenbrenner’s theory.

**Results:**

three categories emerged from the participants’ perceptions: innovation and utility; theory applicability in practice; reinterpreting attitudes of austerity and impatience towards children; disciplining rather than punishing children: dilemmas and challenges. The testimonies highlighted that the Program is innovative, easily accessible, clear in language, and easy to apply in practice for parents/caregivers.

**Conclusion:**

the use of the Program added knowledge and fostered positive attitudes toward the development of the bioecological system in addressing and preventing violence against children.

## Highlights

**(1)** Strengthens sustainable development by protecting children from violence.

**(2)** The program promotes peace and more effective institutions in child protection.

**(3)** Program perception: innovative, applicable to practice, concise, and easy to understand.

**(4)** A relevant tool for professionals working with children and their families.

**(5)** Encourages assertive actions for the prevention of violence against children.

## Introduction

Violence against children negatively impacts their physical, cognitive, and socio-emotional development, leading to severe consequences from childhood through adulthood. Its prevention is listed as one of the goals of the Sustainable Development Goals (SDGs), which, for Brazil, includes “protecting all children and adolescents from abuse, exploitation, trafficking, torture, and all other forms of violence”^(2)^.

The number of reported cases worldwide does not reflect reality due to underreporting, which obscures this global crisis, possibly linked to the fragility of the protection network^(3)^. This situation worsened during the COVID-19 pandemic, as children and adolescents were exposed to increased domestic violence, being forced to spend more time at home and away from protective environments such as schools^(4-5)^.

According to Urie Bronfenbrenner’s bioecological theory, language, symbols, person-to-person and person-context interactions over time (chronosystem), as well as biological, cognitive, emotional, and behavioral aspects, influence human and family development. The interaction process occurs in distinct contexts within systems that are dynamic and interrelated, including the microsystem, mesosystem, exosystem, and macrosystem. Therefore, process, person, context, and time explains the systemic dynamism of the bioecological theory, which can be used to understand that a child who experiences violence will have alterations in their development, world perception, and behavior^(6)^. For example, children who are victims of violence often exhibit aggressive behaviors, increasing the risk of perpetuating such behaviors^(7)^.

Therefore, it is urgent to train those involved in protecting children’s rights to prevent, identify, and intervene early^(3)^. Parental programs can bring short- and long-term benefits^(8)^, constituting a strategy for preventing child maltreatment, although they are scarce in Brazil compared to developed countries like Canada and the United States (USA)^(9-10)^. In Tennessee, USA, at the Vanderbilt University Medical Center, the Play Nicely Program: The Healthy Discipline Handbook was developed by pediatrician Seth Scholer. It is a universal brief intervention program that presents parenting practices to mitigate physical violence against children under eleven years old, consisting of a guide and an interactive educational multimedia tool^(11)^.

The Play Nicely Program: The Healthy Discipline Handbook, translated and cross-culturally adapted for Brazil through methodological steps of independent translations, back-translation, expert evaluation, and pre-testing, was renamed as *Programa Brincar Legal: o guia de disciplina saudável* ^(12)^ *.* It remains associated with Vanderbilt University and is available free of charge for educational and research purposes^(13)^.

The Program presents five steps for addressing acts of aggression against children and behavioral issues, such as the reproduction of acts/symbols of physical violence against peers at school. These steps are I - Teach your child how not to be hurt by others; II - Increase the tools in your toolkit to respond to challenging behavior; III - Decrease exposure to violence and too much media; IV - Show love; V - Be consistent^(11)^. The Program teaches healthy disciplinary practices for parents, teachers, and health professionals, providing 20 options for dealing with children, indicating which actions are good choices and which are not recommended, aiming to prevent punitive attitudes that could lead to other forms of violence, whether psychological, physical, or both^(11)^.

Studies that evaluated pre- and post-intervention using the Program identified a decrease in the use of physical punishment by parents against their children^(8,14-16)^. Additionally, parents reported that after learning from the program, they modified their disciplinary attitudes to include more discussion/explanation, more redirection, more patience/listening, and less anger/yelling^(15-16)^. A study that evaluated pre- and post-training using the program to train health professionals found a significant improvement in their ability to advise parents on appropriate disciplinary strategies, discouraging the use of physical aggression^(17)^. These studies were conducted in other countries, as this study is the first to use the program in Brazil.

Violence against children is characterized by its occurrence during childhood, whether caused by adults or other children, and is commonly classified into types such as physical, psychological, sexual, neglect, and institutional violence^(18)^. Considering this global issue and the absence of free brief intervention programs for the prevention of physical violence against children in this country, this study contributes by presenting a tool to guide healthy disciplinary practices for parents, supporting violence prevention. The Program can be an alternative to assist Brazilian child protection policies and strengthen the fulfillment of the SDGs in educational and health care settings that serve children and their families. In this context, this study aims to understand the perceptions of teachers and health professionals regarding the use of the Play Nicely Program for parents/caregivers in the prevention of violence against children.

## Method

### Study design

This is a qualitative, descriptive, and exploratory study that adhered to the recommendations of the Consolidated Criteria for Reporting Qualitative Research (COREQ).

### Setting

The study was conducted in a municipality in the northern region of Paraná, Brazil, across three institutions: Municipal Early Childhood Education Center (CMEI, acronym in Portuguese), which serves children aged six months to four years old; the Basic Health Unit (UBS, acronym in Portuguese); and the Social Assistance Reference Center (CRAS, its acronym in Portuguese), which assists families in situations of social vulnerability and participates in a federal government program called “*Criança Feliz*,” (Happy Child) aimed at monitoring and strengthening the comprehensive development of children in early childhood, fostering family and community bonds^(19)^.

### Period

The research was conducted from February to April 2022.

### Population

Health and education professionals.

### Selection criteria

Inclusion criteria for education professionals included being a primary school teacher and/or an educator working with children aged one to ten years old. Health professionals were required to be actively working with families that included at least one child within the specified age range, with the parents/caregivers being literate and free from visual or cognitive impairments. Participation in both stages of the research was mandatory for all professionals. Exclusion criteria included being on vacation or leave at the time of the study.

### Participants

Sampling was conducted using convenience sampling, and recruitment was carried out by the principal researcher, who personally met with the coordinators of each team. Subsequently, the coordinator of the CMEI invited the ten teachers from the institution through the WhatsApp application. At the UBS, the coordination suggested approaching the professionals individually, leading to the invitation of nine professionals. At the CRAS, the coordinator held a meeting with the six professionals and explained the study. All professionals agreed to participate, but there was a loss of five participants by the second meeting: one teacher from the CMEI due to contract termination, and at the UBS, participants withdrew because they did not implement the Program guide.

### Data collection

Data collection was conducted solely by the principal researcher, who held a master’s degree and was a faculty member in an undergraduate nursing program at a public university, with experience in focus group techniques. The researcher had no prior connection with the institutions or familiarity with the participants before the study. Social distancing, masks, and hand sanitizer were used as recommended to prevent the spread of COVID-19^(20)^.

Data collection occurred in two sessions with the same participants at each workplace (CMEI, UBS, CRAS) through focus groups. These took place in rooms equipped with rectangular tables, chairs, windows, and minimal external noise. Privacy was ensured by closed doors and well-ventilated areas, with only the participants and the researcher present.

A pilot test was not conducted. During the first meeting, the study’s objective was explained, informed consent was discussed, and signatures were collected, participants individually completed a sociodemographic questionnaire, and a focus group was conducted to explore professionals’ perceptions of violence against children. The program material was also explained and distributed for daily use. This first focus group session was longer, requiring 30 minutes for the program explanation.

During this initial session, participants actively engaged in the discussion. The duration of each session was measured using a chronometer: 47 minutes at CMEI, 67 minutes at UBS, and 59 minutes at CRAS. Participants were instructed to use the Program for 35 days, though no minimum number of sessions was specified. Teachers at CMEI were asked to apply it in the classroom with children, while CRAS and UBS professionals were to guide parents/caregivers on healthy discipline strategies for dealing with disobedience and misbehavior in their children.

The second session took place after the material had been used for the designated period. A focus group was held at each location using a semi-structured script, which included the following questions: What was your experience using the Play Nicely guide? Had you previously heard of healthy discipline practices? If so, tell me more about it. What contributions did the guide bring to your professional practice? How do you perceive your previous knowledge compared to the knowledge gained after using the guide? Were there any difficulties in applying the material? If so, what were they?

The duration of the second focus group was 28 minutes at CMEI, 30 minutes at UBS, and 25 minutes at CRAS. At this stage, five participants dropped out, but the remaining participants actively contributed to the discussion.

In both focus group sessions, the collective interviews were recorded using two audio devices, transcribed in full by a specialized team, and no field diary was used. The transcriptions were made available to participants, but none chose to review or comment on them. The results were not presented to the participants.

### Data treatment and analysis

The data were analyzed and explored using Michel Pêcheux’s discourse analysis, viewed through the lens of Eni Orlandi^(21)^, without the use of software, and discussed considering Urie Bronfenbrenner’s bioecological theory of human development^(6)^. This type of analysis was chosen for its focus on what is not immediately apparent, recognizing that individuals are influenced by ideologies and historical markers, meaning that reality is affected by the symbolic, and people may not be fully aware of how their language and history are influenced.

**Figure 1:**
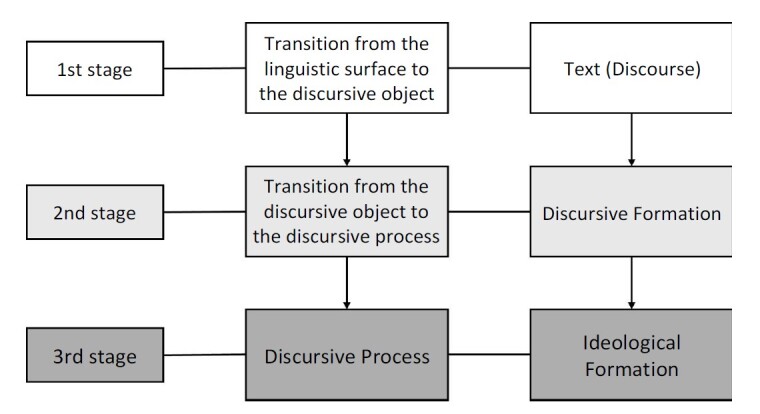
- Flowchart of the analytical framework, adapted from Orlandi^(21)^

The process involved conducting an in-depth analysis of the corpus, that is, the raw material (linguistic surface) resulting from the transcription. This step consists of a linguistic examination of the material, such as identifying who speaks, how they speak, and under what circumstances they speak. Textual markings of synonymy were made during this stage, resulting in 103 codes. From these, the transition was made from the linguistic surface to the discursive object, which is influenced by memories and experiences implicit in the discourse. The third stage involves the transition from the discursive object to the discursive process, which is the movement of understanding and ideological formation, from which categories emerged ^(Figure 1)^. 

### Ethical aspects

The study adhered to ethical standards and was approved by the Research Ethics Committee under opinion number 3,970,750. To protect participants’ identities, they were identified by the letter P followed by a number. Spelling corrections were made, and language fillers were removed without altering the flow of the discourse.

## Results

Out of the 20 participants, all were female, with ages ranging from 23 to 56 years old. The majority were married, had children aged between one and 40 years old, and held a higher education degree. The group included nine teachers, three social workers, three pedagogues, two nurses, one community health worker, one nursing technician, and one psychologist. Their professional experience ranged from three months to 20 years.

Based on the understanding that language is not transparent and that all discourse is shaped by the subject’s history and ideology, the analysis aimed to comprehend how language conveys meaning. Thus, the discursive process revealed that, through the intrinsic and extrinsic knowledge of each person, the use of the Program’s guide was perceived as innovative, useful, applicable, and provided ways to respond to challenging behaviors, along with highlighting dilemmas and challenges.

### Category I – Innovation and utility: applicability of theory in practice:

The guide emerged as a new tool of scientific support and innovation in the workplace. It has the potential to bring practical improvements and complement existing efforts to prevent or combat physical violence against children:

*We have more resources, more material to develop activities, to provide guidance to parents; it is a material that has added to our work* (P1); *It opens up a range of options; we’re no longer limited to just one option; now we have more options to seek out more knowledge* (P18).

For something to be useful, it must satisfy a local reality, offering advantages and proving beneficial. Participants recognized the material’s relevance to their daily reality, noting its comprehensiveness, priorities, content, language, and guidance:

*Simple approaches that are easy to understand, easy to explain to parents in a way that they understand and that does not offend them* (P3).

The guide was highlighted as a set of knowledge and procedures that prioritize guidance for parents/caregivers in their daily actions related to the disciplinary process. Additionally, it provided useful content for developing indirect activities, such as in cases where direct contact with caregivers is not possible, like during a pandemic, allowing it to be used in virtual environments.

*A comprehensive guidance pamphlet was made for everyone, delivered to each family, and then during remote consultations, we provided feedback* (P1).

Using the guide enabled new approaches to addressing challenging situations in the classroom:

*When a child bites or hits another, it was very useful for us to know what to do and how to handle it* (P8); *I used it because my children are two years old; they hit their friends, they bite, so now I know how to approach them, talk to them, and act* (P14).

Seen as a material that enhances daily work, the guide also led to other developments, such as generating new ideas for addressing other types of violence through videos, songs, and the creation and distribution of pamphlets:

*We even got ideas…we did not have the idea to send out activities like that, but after the guide! There is a video, a little song, showing who can touch a child’s body, so we had never thought about it, but after the booklet, we started looking into it* (P4).

One of the Program’s activities involves engaging with people who were parents/caregivers of children about violence, allowing for dialogue and understanding of how past generations were educated:

*I found it interesting; I spoke with a grandmother, and I even thought she would respond by saying she used to use physical punishment too, because that is how she was raised, but no…she did not want to pass that on to the child, the way she was raised* (P19).

Regarding the process of learning and understanding something new, the Program’s guide was considered easy to apply, providing a new perspective on implementing actions in the educational process, a specific and scientifically based source of knowledge on how and when to act:

*The knowledge I had was theoretical. We don’t know how to apply it in practice when faced with a situation. The guide brings more practical suggestions on how to proceed. It gives examples of situations we might encounter and how to handle them. I think that’s the big difference with this material* (P1).

During the professionals’ use of the Program’s guide, one of the actions involved working directly with families in a gentle manner without embarrassing the parents/caregivers, which helped in how to speak, suggest, and guide. Additionally, having the printed material readily available for consultation whenever necessary made it easier to incorporate into daily use.

Some health care professionals discussed the topic of “hitting your child” with parents/caregivers during consultations and were surprised by their responses, stating that they did not punish their children to educate them:

*I was also surprised by the responses from the mothers I interviewed. We always have that mindset that most people are okay with hitting and yelling to educate their children* (P8).

On the other hand, health care professionals might experience contradictions in the parents’ responses compared to the children’s, where a mother claimed not to punish her child, but the child said they were physically punished by her:

*I asked the mother, with the child nearby, and the mother claimed she did not use physical punishment. However, the child responded, “No, mom does hit”. This created a contradiction, as the child was clearly telling the truth. When I asked the mother again, she admitted, “Well, sometimes you have to use physical punishment; there is no other way”.* (P18).

### Category II - Resignifying attitudes of austerity and impatience towards children

Regarding the content, it was emphasized that by guiding parents/caregivers on how to handle their children’s aggression through a lawful approach, and by considering the child as a whole and developing human being, the guide also fosters empathy and humanization:

*The guide specifically explains how to speak in such situations, how to handle them, so it is easier. It provides examples of situations we might encounter and how to proceed* (P1).

The professionals also highlighted certain topics and options offered in the guide, such as disobedience and lying:

*What I often hear is mothers complaining about disobedient children. So, perhaps in the section that talks about disobedience and lying, we could work on more things along those lines* (P5).

The concept of using “no” frequently to interrupt moments of disobedience and establishing clear, concise, and firm rules was also mentioned:

*That discipline of saying “no”, it is not enough to just say “no” to the child, you should explain the reason behind the “no”. I found that very interesting. It is a simple thing that makes a difference* (P8).

The guide provided direction and a clearer approach on how to deal with situations, while also encouraging a more attentive attitude towards parents/caregivers during consultations, thus facilitating dialogue with the child’s caregiver. The strategy of redirecting behavior is illustrated in the following statement:

*There is a part I found interesting, about how to use our body, for example, our hands. If the child hits someone, no, let’s use our hands for something else. If the child kicks a friend, wait a minute, your foot is made for kicking a ball, let’s kick the ball, play soccer, play hopscotch…* (P4).

The interviewees mentioned the strategy of being concerned with others’ feelings, fostering greater empathy:

[…] *she placed as the first option ‘ask your child how the other child feels,’ the only one who responded, the only one who theoretically would go through humanization, empathy, being human* (P16).

Some of the guide’s highlighted content refers to demonstrating love and ensuring quality time with children. In this context, the scarcity of parents’/caregivers’ presence was discussed, often linked to time spent at work, leading to stress and strained relationships.

The visibility provided by the guide regarding the importance of caregivers having a consistent language with the child and knowing how to approach them without frightening them was emphasized, as many parents/caregivers “yell” during discipline, which can lead to unsuccessful discipline and affect the child’s emotional security.

The professionals also highlighted the control of daily screen time and the inclusion of sports activities in the child’s educational process:

*I thought it was good what they said about the TV, the tablet, that we need to set limits, we know it is better, and the guide also talks about sports as an option. Very good, I found it interesting* (P15).

### Category III – Disciplining the child, not punishing: dilemmas and challenges

The interviewees also discussed the dilemmas they faced in the educational process, particularly the challenges in establishing alternative disciplinary rules, such as grounding a child by restricting them to their room or taking away their cellphone, which is, using other means to educate:

*The mother says she talks a lot, but she always punishes. She does not hit, but she grounds the child and makes her reflect. Hitting is against her principles, but she usually confines her to her room, takes away her cellphone, in short, she does something* (P19).

The issue of “hitting in certain situations” was still mentioned as a dilemma, particularly in situations that depend on the sense of justice and injustice, defense and attack, which relate to personal and family values:

*If another child hits my daughter, I would hit my daughter, because she must have done something, so I would educate her by hitting her* (P20).

There were also uncertainties about whether or not to retaliate when an aggression occurs:

*A mother told me that she encourages her daughter to retaliate because her older son, who is 15 years old, was a “punching bag” at school, and she does not want the same thing to happen to her daughter, who is now 8 years old, so she teaches her that whatever someone does to her, she should fight back* (P19).

Another dilemma in the parental education process was how to maintain patience, as education is a long and repetitive process since children do not understand everything at once:

*I think the difficulty is always having to repeat the same thing because sometimes it is just a word, like with my son who is not used to listening, so you have to keep repeating the same thing until they understand* (P9).

Regarding challenges, the professionals reported difficulties in addressing the topic of violence during consultations before explaining the guide, as well as in intervening when a discussion about non-recommended disciplinary practices began.

For some professionals who voluntarily work directly with families participating in a social program, there was hesitation in fully utilizing the guide at the outset, fearing that families might withdraw from the program. It is a delicate topic, and depending on the approach, parents/caregivers might perceive it as an invasive critique of how they educate and punish their children:

*We cannot interfere too much, you know? This pamphlet was made broadly, for everyone, as guidance. If the family feels that we are trying to meddle or say they are doing something wrong, they do not like it and say: “Oh, I do not want you here anymore”* (P1).

On the other hand, some interviewees also mentioned the difficulty of remembering everything at the time of application and not being able to fully apply what they learned in practice:

*We cannot remember everything, but we can continue applying some of it; we have to read and reread the book again; we will always be consulting it* (P9).

Despite highlighting these dilemmas, they expressed that they liked the guide, considering it “helpful” and “interesting”, and found it valuable for guiding parents/caregivers. There was also positive acceptance from the parents/caregivers involved during the professionals’ use of the guide, and they showed a great deal of interest in the material.

## Discussion

Programs that assist parents in fulfilling their parental roles are fundamental to achieving the goals of child protection services, recommended as key strategies for preventing child abuse^(10,22)^. They also promote healthy parenting practices, help mitigate violence, and strengthen SDG 16, which aims to promote peace, justice, and effective institutions^(2)^.

In Brazil, children in situations of social inequality face risks of violence due to factors such as low socioeconomic status, lack of education, food, and health care^(23)^. In this study, the Program proved to be innovative in the daily work environment, with its easy-to-understand language facilitating its use with vulnerable families served by the CRAS. A clinical trial in Nashville, USA, also showed similar results with low-income families^(15)^.

There was learning about the types of violence and how to address them, redirecting aggressive behaviors into positive ones, such as using the foot to kick a ball instead of a peer. Acts like biting, pushing, hair-pulling, and hitting are related to immaturity and should be shaped for the child’s emotional regulation^(24)^. Parents, as behavior modulators, should assist in the child’s self-regulation^(25)^. Teaching them kindly about their actions increases the chances of educational success^(11)^, directly impacting relationships within the microsystem^(6)^, promoting a change that can lead to healthy behaviors. Disobedience, lying, yelling, biting, and aggression can evolve into antisocial behavior disorders, interpersonal violence, and criminalization during adolescence^(26-27)^.

The professionals in this study emphasized the importance of explaining the “why not”, understanding that the child is developing and needs this dialogue. This educational attitude aligns with the literature, which recommends using brief responses during the child’s disciplinary moment, given that their brain development is gradual, and the prefrontal cortex, responsible for cognitive control behaviors, only reaches maturity in adulthood^(28)^. A specific and simple statement of the expected action should be provided, clearly stating what to do rather than what not to do^(11,29)^.

The professionals highlighted the importance of “asking how the other child feels” in cases of aggression between children, teaching empathy. Empathy is crucial for self-regulation and healthy socioemotional development^(25)^. Parents should have consistent parenting styles, as different levels of control can negatively impact the development of empathy and foster antisocial behaviors^(30-31)^. According to Bronfenbrenner, in the microsystem, frequent and continuous relationships are essential for healthy development, reflecting what is learned in the mesosystem^(6)^.

The amount of time spent on media was a topic in the guide that caught the attention of professionals and is directly associated with difficulties in emotional self-regulation during child development^(32)^. Exposure to violent video games increases the risk of desensitization to violence, can increase aggressiveness, and decrease prosocial behavior^(33)^. In this regard, other researchers have also shown that problematic use of social media is directly related to low levels of closeness between parents and children^(34)^. Furthermore, in the bioecological theory of human development, all systems influence the formation of a person. They are shaped by the environment where the child is located and interacts, whether directly or indirectly^(6)^.

The use of the Play Nicely Program guide enhanced and contributed to solidifying content, which was put into practice in the daily routines of professionals in different child and family care settings. This guide was also considered practical and easily applicable by Colombian parents, and there was a significant reduction in the use of physical punishment and a decrease in the belief that punishment is the best alternative for educating and controlling children’s behavior^(8)^.

The dilemmas highlighted by professionals related to the process of disciplining children, as teaching children how to behave is a continuous process that occurs over months and years, requiring a great deal of patience. In this context, the literature shows that the way of educating is influenced by experiences, socio-historical-cultural context, ideology, and environment^(6)^. Amidst the complexity of educating a child, it is prudent to consider the transmission of healthy attitudes to avoid perpetuating violent behavior. To this end, positive parenting approaches can be used to strengthen protective factors^(35)^.

Regarding the difficulties encountered by professionals in approaching parents/caregivers to discuss the guide, these challenges may be related to the need for greater familiarity and skill with the topic. Ideally, this training should begin during undergraduate education and continue through ongoing professional development in the institutions where they work. Violence against children remains one of the greatest challenges in our society, and the implementation of various actions to break the cycle of violence is utterly needed.

The growing intolerance toward violence was reflected in the results of a national survey, which identified an increase in reports to the *Disque 100* hotline in 2021. The hotline recorded 186,862 cases of violations of children’s rights in domestic environments, including mistreatment, exposure, bodily harm, psychological torture, threats, femicide, bullying, and other forms of abuse^(23)^.

Given the scarcity of parental programs in the country aimed at preventing violence against children^(23)^, the contribution of this study is noteworthy. Considering the insights of education and health professionals on a feasible Program guide for parents/caregivers, there is potential to mitigate violence against their children and, consequently, adjust intergenerational attitudes that still tolerate physical, psychological, and other forms of punishment as means of educating or disciplining a child.

Additionally, new possibilities need to be offered to interrupt the cycles of violence that have perpetuated across generations, thereby promoting a culture of peace as established in the sustainable development goals.

The study’s limitations include the need for the Play Nicely Program to be used in other settings, with new research designs for deeper exploration and assessment of its effectiveness. The study of parents/caregivers in the context of their homes could serve as an example, although it was not possible at the time of this study due to the restrictions related to the COVID-19 pandemic.

## Conclusion

The Play Nicely Program was viewed by professionals as an innovative, useful, practical tool with easy-to-understand language. It enhanced knowledge, improved practices, and facilitated dissemination among parents/caregivers in both education and health care settings. It stood out for addressing the topic indirectly, thus avoiding discomfort and promoting a broad educational perspective. The main challenge was remembering and applying all the content, a challenge that can be overcome by integrating the guide into daily routines.

Having a brief and comprehensive program enables professionals to gain greater confidence in their actions, contributing to more effective institutions in child protection, such as in primary care, during pediatric consultations, and in school health services, with the aim of preventing violence.

Consequently, it benefits the general population and children, as healthy parenting practices can positively influence the development of the bioecological system. Thus, the Program also supports sustainable development by protecting children from violence, promoting peace, and strengthening effective institutions.
