# Supplementary material for: Play Nicely Program in the prevention of violence against children: strengthening sustainable development
Source: Rev Lat Am Enfermagem. 2025 Jan 27;33:e4434. doi: 10.1590/1518-8345.7320.4434 (PMC11774143; doi:10.1590/1518-8345.7320.4434)
Supplement: Translation

# Translation: ***Programa Brincar Legal*** para prevenir la violencia contra los niños: fortalecer el desarrollo sostenible^*^

**DOI:** 10.1590/1518-8345.7320.4433

**Authors:** Janaina Recanello Begui, Andressa Larissa Dias Müller de Souza, Naiara Barros Polita, Maria de Fátima Garcia Lopes Merino, Adriana Valongo Zani, Rosângela Aparecida Pimenta

**Affiliations:** 1 Universidade Estadual do Norte do Paraná, Departamento de Enfermagem, Bandeirantes, PR, Brasil.; 2 Universidade Estadual de Londrina, Londrina, PR, Brasil.; 3 Universidade Estadual de Londrina, Departamento de Enfermagem, Londrina, PR, Brasil.; 4 Universidade Estadual de Maringá, Departamento de Enfermagem, Maringá, PR, Brasil.

**Keywords:** Protección a la Infancia, Maltrato a los Niños, Responsabilidad Parental, Crianza del Niño;Atención Primaria de Salud, Desarrollo Sostenible

## Abstract

**Objetivo:**

comprender la percepción de docentes y profesionales de la salud sobre el uso del *Programa Brincar Legal* para padres/cuidadores para prevenir la violencia contra los niños.

**Método:**

estudio cualitativo, descriptivo y exploratorio realizado por medio de tres grupos focales con veinte docentes de educación básica y profesionales de atención primaria de salud que aplicaron el Programa para padres/cuidadores en 2022. El análisis del discurso francés guió el análisis de los datos a la luz de la teoría de Urie Bronfenbrenner.

**Resultados:**

a partir de la percepción de los participantes surgieron tres categorías: innovación y utilidad: aplicabilidad de la teoría en la práctica; resignificar actitudes severas e impacientes hacia los niños; disciplinar al niño y no castigarlo: dilemas y desafíos. Las declaraciones revelaron que el Programa es innovador, de rápido acceso, con lenguaje claro y fácil de aplicar en la práctica para padres/cuidadores.

**Conclusión:**

el uso del Programa aportó conocimiento y promovió actitudes positivas para el desarrollo del sistema bioecológico para enfrentar y prevenir la violencia contra los niños.

## Destacados

**(1)** Fortalece el desarrollo sostenible, protegiendo a los niños de la violencia.

**(2)** El programa promueve la paz e instituciones más efectivas en la protección infantil.

**(3)** Percepción sobre el Programa: innovador, aplicable a la práctica, breve y con lenguaje simple.

**(4)** Es una herramienta importante para los profesionales que trabajan en el cuidado de los niños y sus familias.

**(5)** Promueve acciones asertivas para prevenir la violencia contra los niños.

## Introducción

La violencia contra los niños tiene un impacto negativo en su desarrollo físico, cognitivo y socioemocional, y genera graves consecuencias desde la infancia hasta la edad adulta^(1)^. La prevención de la misma figura entre las metas de los Objetivos de Desarrollo Sostenible (ODS), que, para Brasil, prevé “proteger a todos los niños y adolescentes del abuso, la explotación, la trata, la tortura y todas las demás formas de violencia”^(2)^.

El número de casos reportados en todo el mundo no refleja la realidad, debido al subregistro que oculta la crisis global que, probablemente, está ligada a la fragilidad de la red de protección^(3)^. La situación se agravó durante la pandemia de COVID-19, dado que expuso a niños y adolescentes a una mayor violencia doméstica, porque se vieron obligados a pasar más tiempo en casa y lejos de ambientes protectores, como las escuelas^(4-5)^.

Según la teoría bioecológica de Urie Bronfenbrenner, el lenguaje, los símbolos, la interacción persona-persona y persona-contexto, a lo largo del tiempo (cronosistema), así como aspectos biológicos, cognitivos, emocionales y conductuales, influyen en el desarrollo humano y familiar. El proceso de interacción se da en diferentes contextos presentes en sistemas que son dinámicos y están interrelacionados, a saber: microsistema, mesosistema, exosistema y macrosistema. Por lo tanto, el proceso, la persona, el contexto y el tiempo explican el dinamismo sistémico de la teoría bioecológica, que se puede utilizar para comprender que los niños que sufren violencia tendrán cambios en el desarrollo, la percepción del mundo y la conducta^(6)^. Por ejemplo, las víctimas de violencia en la infancia suelen manifestar conducta agresiva, lo que aumenta el riesgo de perpetrar la misma^(7)^.

Por lo tanto, es urgente capacitar a los involucrados en la protección de los derechos de los niños para prevenir, identificar e intervenir tempranamente^(3)^. Los programas parentales pueden traer beneficios a corto y largo plazo^(8)^, son una estrategia para prevenir el abuso, aunque son escasos en Brasil en comparación con países desarrollados como Canadá y Estados Unidos (EE.UU.)^(9-10)^. En Tennessee, EE.UU., en el *Vanderbilt University Medical Center*, el pediatra Seth Scholer desarrolló el *Play Nicely Program: The Healthy Discipline Handbook*, un programa universal de intervención breve que presenta prácticas parentales para reducir la violencia física contra niños menores de once años, que consistente en una guía y multimedia interactiva educativa^(11)^.

La guía del *Play Nicely Program: The Healthy Discipline Handbook*, traducida y adaptada transculturalmente a Brasil, mediante pasos metodológicos de traducciones independientes, retrotraducción, evaluación por expertos y pretest, se denominó *Programa Brincar Legal: o guía de disciplina saudável* ^(12)^, sigue vinculada a la Universidad Vanderbilt y está disponible de forma gratuita con fines educativos y de investigación^(13)^.

El Programa presenta cinco pasos a seguir ante actos de agresión contra el niño y problemas de conducta, como la reproducción de actos/símbolos de violencia física contra sus compañeros en la escuela, que son: I-Enséñale a tu hijo a no ser una víctima; II-aprende maneras de actuar frente a comportamientos desafiantes; III-reduce la exposición a la violencia y a los medios de comunicación en exceso; IV-demuestra amor; V-sé firme^(11)^. Enseña prácticas disciplinarias saludables para padres, profesores y profesionales de la salud, da 20 ejemplos de opciones para lidiar con los niños, indica qué acciones son buenas opciones y cuáles no son recomendables, con el objetivo de evitar actitudes punitivas que generen otros tipos de violencia como la psicológica, la física o ambas^(11)^.

Estudios que evaluaron la preintervención y la postintervención utilizando el Programa identificaron que los padres redujeron el uso del castigo físico contra los niños^(8,14-16)^. Además, los padres afirmaron que, después de aprender del programa, cambiaron sus actitudes disciplinarias, recurrieron más a la conversación/explicación, la redirección, la paciencia/escucha y menos al enojo/gritos^(15-16)^. Un estudio que evaluó el pre y post entrenamiento utilizando el programa de capacitación de profesionales de la salud identificó una mejora significativa en la capacidad para asesorar a los padres sobre estrategias disciplinarias adecuadas, que desalientan el uso de la agresión física^(17)^. Estos estudios se realizaron en otros países, ya que el presente estudio es el primero que utiliza el programa en Brasil.

La violencia contra los niños se caracteriza por ocurrir en la infancia, ya sea causada por adultos o por otros niños, y se clasifica como física, psicológica, sexual, negligencia e institucional^(18)^. Considerando que se trata de un problema a nivel mundial y que no hay programas gratuitos de intervención breve en Brasil para prevenir la violencia física contra los niños, este estudio contribuye al presentar una herramienta que puede orientar a los padres para que adopten prácticas disciplinarias saludables y apoya la prevención de la violencia. El Programa puede ser una alternativa que contribuya a las políticas brasileñas de protección de la infancia y fortalezca el cumplimiento de los ODS en los espacios educativos y de atención para la salud que atienden a los niños y sus familias. Por lo tanto, este estudio tiene como objetivo comprender la percepción de docentes y profesionales de la salud sobre el uso del *Programa Brincar Legal* para padres/cuidadores para prevenir la violencia contra los niños.

## Método

### Diseño del estudio

Se trata de un estudio cualitativo, descriptivo y exploratorio, que cumplió con las recomendaciones del *Consolidated Qualitative Research Reporting Criteria* (COREQ).

### Escenario

Estudio realizado en un municipio del norte del Estado de Paraná, Brasil, en tres instituciones: Centro Municipal de Educación Infantil (CMEI), que atiende a niños de entre seis meses y cuatro años; Unidad Básica de Salud (UBS) y; Centro de Referencia de Asistencia Social (CRAS), que atiende a familias en situación de vulnerabilidad social y está incluido en un programa del gobierno federal denominado “*Criança Feliz*”, cuyo objetivo es monitorear y fortalecer el desarrollo integral de los niños en la primera infancia, estimulando el vínculo familiar y comunitario^(19)^.

### Período

La investigación se llevó a cabo de febrero a abril de 2022.

### Población

Profesionales de la salud y la educación.

### Criterios de selección

Los criterios de inclusión para profesionales de la educación fueron ser docente de educación básica y/o pedagogo(a), que trabaje con niños de entre uno y diez años. Para los profesionales de la salud, deben trabajar directamente con familias compuestas por al menos un menor dentro de la franja etaria mencionada anteriormente, los padres/cuidadores deben estar alfabetizados y no tener discapacidad visual o cognitiva. Todos los profesionales deben participar en ambas etapas de la investigación. Los criterios de exclusión para los profesionales fueron estar de vacaciones o licencia.

### Participantes

El muestreo fue por conveniencia y el reclutamiento fue realizado por la investigadora principal, quien habló personalmente con la coordinación de cada equipo. Posteriormente, la coordinadora del CMEI, mediante la aplicación *WhatsApp*, invitó a los diez docentes de la institución. En la UBS, la coordinación sugirió acercarse directamente a los profesionales, nueve profesionales fueron invitados individualmente. En el CRAS, la coordinadora tuvo una reunión con los seis profesionales y les informó sobre el estudio. Todos los profesionales aceptaron participar, pero en la segunda reunión hubo una pérdida de cinco personas: en el CMEI un profesor abandonó porque finalizó su contrato, en la UBS se retiraron porque no aplicaron la guía del Programa.

### Recolección de datos

La recolección de datos fue realizada solamente por la investigadora principal. Cuando se llevó a cabo el estudio, ella tenía una maestría, se desempeñaba como docente en la carrera de enfermería de una universidad pública, tenía experiencia en técnicas de grupos focales, no tenía ningún tipo de vínculo con las instituciones del estudio, ni conocía a los participantes antes de la investigación. Se cumplió con todas las medidas recomendadas para prevenir la propagación y el contagio por COVID-19, distanciamiento, uso de mascarillas y alcohol en gel^(20)^.

La recolección de datos se realizó en dos momentos distintos con los mismos participantes, en cada lugar de trabajo (CMEI, UBS, CRAS), a través de un grupo focal. Se llevaron a cabo en salas que contaban con mesas rectangulares con sillas, ventanas y poco ruido exterior. La privacidad se logró cerrando las puertas, aun así, el ambiente estaba aireado, y contando solo con la presencia de los participantes y la investigadora.

No se realizó ninguna prueba piloto. Las actividades de la primera reunión fueron: explicación del objetivo del estudio; explicación del formulario de consentimiento libre e informado; recolección de firmas; cumplimentación individual de un cuestionario sociodemográfico; realización de un grupo focal para comprender la percepción de los profesionales sobre la violencia contra los niños; explicación y entrega de material para uso del Programa en la rutina diaria. En el primer encuentro, el grupo focal duró más, ya que se requirieron 30 minutos para explicar el programa.

En el primer momento, el grupo participó activamente de la discusión, el tiempo fue medido con un cronómetro, fueron 47 minutos en el CMEI, 67 minutos en la UBS y 59 minutos en el CRAS. Se les indicó a los participantes que utilizaran el programa durante 35 días, pero no se determinó un número mínimo de citas. Se les solicitó a los docentes que lo aplicaran en el aula con niños del CMEI, y a los profesionales del CRAS y de la UBS que orientaran a los padres/cuidadores sobre estrategias de disciplina saludable para enfrentar las actitudes de desobediencia e indisciplina de sus hijos.

El segundo momento se produjo después de finalizar el periodo de uso del material. En cada sede se realizó un grupo focal utilizando un formulario semiestructurado, que contenía las siguientes preguntas: ¿qué significó para ti utilizar la guía *Brincar Legal*? ¿Has oído hablar alguna vez de disciplinas saludables? Si es así, cuéntame más al respecto. ¿Qué aportes hizo la guía a tu práctica profesional? ¿Cuál es tu percepción sobre tus conocimientos previos y los adquiridos después de utilizar la guía? ¿Tuviste dificultades para aplicar el material? Si es así, ¿cuáles fueron?

El grupo focal tuvo una duración de 28 minutos en el CMEI, 30 minutos en la UBS y 25 minutos en el CRAS. En esta etapa se perdieron cinco participantes, pero los demás colaboraron activamente en la discusión.

En los dos momentos de los grupos focales, las entrevistas colectivas fueron grabadas por dos dispositivos de audio, transcritas completamente por un equipo especializado y no se utilizó el diario de campo. Las transcripciones se pusieron a disposición de los participantes, pero ninguno quiso leerlas para hacer comentarios o correcciones. No se les presentaron los resultados a los participantes.

### Procesamiento y análisis de datos

Los datos fueron analizados y explorados a partir del análisis del discurso de Michel Pêcheux bajo la mirada de Eni Orlandi^(21)^, sin el uso de *software*, y discutidos a la luz de la teoría bioecológica del desarrollo humano de Urie Bronfenbrenner^(6)^. Se eligió este tipo de análisis porque considera lo que no es transparente, dado que el sujeto está dotado de ideologías y marcas históricas, de modo que lo real se ve afectado por lo simbólico y la persona no tiene control ni conciencia de cómo se ven afectados su lenguaje y su historia.

Se dessuperficializó el *corpus*, es decir, el material bruto (superficie lingüística) resultante de la transcripción. Esta etapa consiste en el análisis lingüístico del material, como, por ejemplo, quién lo dice, cómo lo dice y en qué circunstancias lo dice, se realizaron marcas textuales de sinonimia que generaron 103 codificadores. A partir de ellos, se pasó de la superficie lingüística al objeto discursivo, que es afectado por recuerdos y experiencias que están implícitas en el discurso. La tercera etapa es el pasaje del objeto discursivo al proceso discursivo, es decir, el movimiento de comprensión y formación ideológica, en el que surgieron las categorías ^(Figura 1)^.

**Figura 1:** - Diagrama de flujo del dispositivo analítico, adaptado de Orlandi^(21)^

### Aspectos éticos

El estudio cumplió con las normas éticas y fue aprobado por el Comité de Ética en Investigación con dictamen número 3.970.750. Para no exponer a los participantes, se los identificó con la letra P y un número, se realizaron correcciones ortográficas y se eliminaron errores de lenguaje, sin alterar el flujo.

## Resultados

Los 20 participantes eran del sexo femenino, tenían entre 23 y 56 años, la mayoría estaba casada, con hijos entre uno y 40 años y había completado estudios superiores, nueve de ellas eran docentes, tres trabajadoras sociales y tres pedagogas, dos enfermeras, una era agente comunitario de salud, una técnica en enfermería y una psicóloga. El tiempo de profesión oscilaba entre tres meses y 20 años.

Partiendo de la concepción de que el lenguaje no es transparente y de que todo discurso es interpelado por la historia y la ideología del sujeto, se procuró, en el proceso de análisis, entender que el lenguaje tiene sentido. De esa forma, el proceso discursivo mostró, a través del conocimiento intrínseco y extrínseco de cada persona, la percepción de que el uso de la guía del Programa representó innovación, utilidad, aplicabilidad, formas de actuar ante conductas desafiantes, dilemas y desafíos.

### Categoría I – Innovación y Utilidad: aplicabilidad de la teoría en la práctica

La guía se presentó como una nueva herramienta de base científica e innovadora en el lugar de trabajo. Tiene el potencial de aportar mejoras prácticas y sumarse a las que ya existen para prevenir o combatir la violencia física contra los niños:

[…] *tienes más cosas, más material para preparar actividades, para preparar orientaciones para los padres, es un material que le aporta algo nuevo a nuestro trabajo* (P1)*; se abre una gama de opciones, no nos circunscribimos a una sola opción, ahora tenemos más opciones para buscar más conocimiento* (P18).

Para que algo se vuelva útil debe satisfacer las necesidades de una realidad local, tener ventajas y ser beneficioso. Los participantes notaron el uso que podían darle al material en su trabajo diario, el alcance y las prioridades, el contenido, el lenguaje y el asesoramiento:

*Enfoques fáciles de entender, sencillo, lenguaje simple que podemos transmitirle a los padres de forma que ellos entiendan y que no se ofendan* (P3).

La guía se destacó como un conjunto de saberes, de procedimientos que priorizan el asesoramiento para que los padres/cuidadores los apliquen en la vida diaria y que están relacionados con el proceso disciplinario. Además, tenía contenido útil para desarrollar una actividad indirecta, es decir, en los casos en los que no se podía establecer contacto directo con los cuidadores, como en la pandemia, se puede utilizar para brindar atención en entornos virtuales.

*Se elaboró un folleto orientativo, integral, para todos, que se le entreg*ó *a cada familia, y después realizamos las consultas remotas y fuimos teniendo una retroalimentación* (P1).

El uso de la guía abrió nuevos caminos, nos orientó para afrontar situaciones desafiantes del día a día en el aula:

[…] *cuando un niño muerde, golpea a otro, fue muy útil para saber qué hacer, cómo debemos afrontarlo* (P8); *la usé, porque mis hijos tienen dos años, les pegan a sus amiguitos, los muerden, entonces, ahora sé cómo afrontarlo, hablar, actuar* (P14).

Se lo considera un material que aporta algo nuevo al trabajo diario, la guía presentó otras opciones, como nuevas ideas para el abordaje de otros tipos de violencia, a través de videos, música, elaboración y creación de folletos:

*Incluso nos dio ideas... no pensábamos enviar actividades como esta, ¡pero después de la guía! Hay un vídeo que muestra, una pequeña canción, que muestra quién puede tocar el cuerpo del niño, nunca lo habíamos pensado, después del folleto empezamos a hacerlo* (P4).

Una de las actividades del Programa también es acercarse a las personas que fueron padres/cuidadores de niños y hablar sobre la violencia, eso nos permitió dialogar y comprender la forma de educar de las generaciones pasadas:

[…] *me pareció interesante, hablé con una abuela y hasta pensé que ella iba a responder que había que golpearla, porque ella recibió esa educación, pero no... ella no quería criar a la niña de la misma forma que la criaron a ella* (P19).

En lo que respecta al proceso de aprender y aprehender, se consideró que la guía del Programa es fácil de aplicar, que brinda una nueva perspectiva para implementar acciones en el proceso educativo, una fuente de conocimiento específica y con base científica sobre cómo y cuándo realizar ciertas acciones:

*El conocimiento que tenía era teórico. No sabía cómo poner eso en práctica ante una situación real. La guía proporciona, de una forma más práctica, sugerencias sobre cómo hacerlo. Da ejemplos de situaciones que podemos estar viviendo y cómo manejarlas. Creo que esta es la gran diferencia con este material* (P1).

Una de las acciones que realizaron los profesionales cuando usaron la Guía del Programa fue trabajar directamente con las familias de manera gentil y sin avergonzar a los padres/cuidadores, los ayudó para saber cómo hablarles, hacer sugerencias y asesorarlos. Además, el acceso inmediato al material impreso para consultarlo cuando fuera necesario facilitó el uso diario.

Algunos profesionales de la salud hablaron con los padres/cuidadores durante la consulta sobre “golpear a su hijo”, se sorprendieron por las respuestas de que no castigaban a sus hijos para educarlos:

[…] *a mí también me sorprendieron las respuestas de las madres que entrevisté. Siempre pensamos que la mayoría de la gente coincide en que hay que pegarle y gritarle al niñopara educarlo* (P8).

Por otro lado, el profesional de la salud puede experimentar una contradicción en la respuesta de la madre en perjuicio del niño, cuando ella afirmó que no lo castigaba y su hijo dijo que ella lo castigaba físicamente:

[…] *le pregunté a la madre y el niño estaba cerca, y entonces la madre dijo que no le pegaba, y el niño dijo “ella me pega”. Entonces, hay una contradicción, es decir, el niño realmente está diciendo la verdad. Entonces le pregunté y me dijo: “ah, de vez en cuando hay que pegarle, no queda otra, no hay forma”* (P18).

### Categoría II - Resignificar actitudes severas e impacientes hacia los niños

En cuanto al contenido, se destacó que, al asesorar a los padres/cuidadores sobre cómo proceder en casos de agresión por parte de sus hijos desde un enfoque amable, y al considerar al niño como un ser humano integral y en desarrollo, también favorece la empatía y humanización:

*La guía explica específicamente cómo hablar en una situación así, cómo manejarse para que sea más fácil, da ejemplos de situaciones que podemos enfrentar y cómo proceder* (P1).

Los profesionales también destacaron algunos temas y algunos consejos que brinda la guía, como la desobediencia y la mentira:

*Escucho mucho que la madre se queja de que el niño es desobediente.Entonces, tal vez en el punto que habla de la desobediencia, de que el niño miente, podamos trabajar más al respecto* (P5).

También se mencionó entender el uso del “no” como recurso frecuente para interrumpir los momentos de desobediencia del niño y establecer reglas claras, breves y firmes:

[…] *que la disciplina del no, no basta con decirle “no” al niño, hay que explicarle por qué no. Me pareció muy interesante. Es algo sencillo que marca la diferencia* (P8).

La guía dio más consejos sobre cómo afrontar las situaciones, además fomenta que se observe más atentamente a los padres/cuidadores durante la consulta, y facilita el diálogo con el cuidador del niño. La estrategia de redirigir la conducta se puede ver en el siguiente enunciado:

[…] *hay una parte que me pareció interesante, es cómo utilizar nuestro cuerpo, por ejemplo, nuestras manos. El niño fue allí y golpeó, no, usemos nuestra manito para hacer otra cosa. Luego le dio una patada a un amigo, no, para, tu pie está hecho para patear la pelota, pateemos la pelota, juguemos al fútbol, a la rayuela…* (P4).

Los entrevistados propusieron la estrategia de preocuparse por los sentimientos de los demás, demostrando mayor empatía:

[…] *planteó como primera opción “pregúntale a tu hijo cómo se siente el otro niño”, la única que respondió, la única que teóricamente pasaría por la humanización, la empatía, ser humano* (P16).

Algunos contenidos destacados en la guía se refieren a demostrar amor y garantizar tiempo de calidad con sus hijos. En este sentido, se abordó la falta de presencia de los padres/cuidadores, muchas veces relacionada con el tiempo que pasan en el trabajo, que genera estrés y compromete las relaciones.

Se destacó la visibilidad que brindó la guía sobre la importancia de que los cuidadores tengan el mismo lenguaje que el niño y sepan acercarse sin asustarlo, ya que muchos padres/cuidadores “gritan” durante el acto disciplinario, lo que puede provocar que fracase la disciplina y afectar tu seguridad emocional.

Los profesionales también destacaron el control del tiempo diario al que están expuestos a los medios de comunicación y las prácticas deportivas en el proceso educativo del niño:

[…] *me pareció genial lo de la televisión, lo de la* tablet*, que hay que poner un límite, uno sabe que es mejor, y la guía habla también de las prácticas deportivas como opción. Es muy bueno, me pareció interesante* (P15).

### Categoría III – Disciplinar al niño y no castigarlo: dilemas y desafíos

Los entrevistados también plantearon los dilemas que tuvieron en el proceso educativo, las dificultades para establecer otras reglas disciplinarias, como dejarlo en la habitación, quitarle el celular, es decir, cómo utilizar otros medios para educar:

*La madre dice que habla mucho, pero siempre castiga. No lo golpea, pero lo castiga y lo hace reflexionar. Golpearlo es contraproducente, normalmente lo deja en el cuarto, le quita el celular, en fin, algo hace* (P19).

Se menciona como un dilema “pegarle en determinadas situaciones”, situaciones que dependen del sentido de justicia e injusticia, de defensa y ataque, que están relacionadas con valores personales y familiares:

*[…] si otro niño golpea a mi hija, yo le pegaría a ella, porque algo hizo, entonces la voy a educar golpeándola* (P20).

También hubo dudas sobre si defenderse o no cuando ocurre una agresión:

*Una madre me dijo que anima a su hija a defenderse, porque su hijo mayor, de 15 años, era un “saco de boxeo” en la escuela y ella no quiere que le pase lo mismo a su hija que hoy tiene 8 años, entonces le enseña que cuando le hagan algo, ella se la devuelva* (P19).

Otro dilema en el proceso de educación de los padres fue cómo tener paciencia, dado que la educación es un proceso largo y repetitivo porque el niño no entiende todo de entrada:

[…] *creo que la dificultad es estar siempre ahí insistiendo, porque a veces es una palabra, lo mismo pasa con mi hijo que no está acostumbrado a oír, entonces hay que seguir insistiendo hasta que entienda* (P9).

Con respecto a esos desafíos, los profesionales mencionaron que enfrentaron dificultades durante la atención para abordar el tema de la violencia antes de explicarle la guía y también para intervenir cuando comienza una discusión sobre prácticas disciplinarias no recomendadas.

Algunos profesionales que trabajan directamente con familias que participan en un programa social de forma voluntaria, al principio tuvieron miedo de utilizar la guía completa por temor a que las familias se fueran. Este es un tema delicado y, dependiendo del enfoque, los padres/cuidadores pueden interpretarlo como una actitud invasiva sobre la forma de educar y castigar a sus hijos:

*No podemos interferir demasiado, ¿sabes? Esta información (folleto) fue creada de manera integral, para todos, a modo de orientación. Si la familia se da cuenta que estamos tratando de criticarlos o decirles que están haciendo algo mal, no les gusta y dicen: “ay, ya no te quiero aquí”* (P1).

Por otro lado, algunos entrevistados también mencionaron que tenían dificultades para recordar todo a la hora de aplicarlo y que no podían aplicar lo aprendido en la práctica:

[…] *no podemos recordar todo, pero recordar un poco nos permite seguir aplicándolo, el libro lo tenemos que leer, releer y volver a leer, siempre lo consultamos* (P9).

Incluso frente a estos dilemas, dijeron que les gustó la guía, la consideraron “genial” e “interesante”, válida para asesorar a los padres/cuidadores. Los padres/cuidadores participantes también demostraron buena aceptación cuando la usaron los profesionales y mostraron mucho interés en el material.

## Discusión

Los programas que ayudan a los padres a desempeñar el rol parental son fundamentales para cumplir los objetivos de los servicios de protección infantil, se los recomienda como estrategias importantes para prevenir el abuso infantil^(10,22)^. También fomentan prácticas parentales saludables, ayudan a reducir la violencia y fortalecen el objetivo 16 de los ODS, cuyo objetivo es promover la paz, la justicia y las instituciones eficaces^(2)^.

En Brasil, los niños en situaciones de desigualdad social enfrentan riesgos de violencia debido a factores como el bajo nivel socioeconómico, la falta de educación, alimentación y salud^(23)^. En este estudio, el Programa demostró ser innovador para el trabajo diario, el lenguaje simple favoreció el uso con familias vulnerables que reciben atención en el CRAS. Un ensayo clínico en Nashville, EE.UU., también arrojó resultados similares con familias de bajos ingresos^(15)^.

Se aprendió sobre los tipos de violencia y cómo abordarlos, redirigiendo conductas agresivas hacia otras positivas, como usar el pie para patear una pelota en lugar de a un amigo. Actos como morder, empujar, tirar del cabello y golpear están relacionados con la inmadurez y deben ser moldeados para favorecer la regulación emocional del niño^(24)^. Los padres, como moduladores de la conducta, deben contribuir a la autorregulación del niño^(25)^. Enseñarles amablemente qué actitudes tomar aumenta las posibilidades de éxito educativo^(11)^, afecta directamente la relación del microsistema^(6)^, promueve un cambio que puede genera acciones saludables. Por lo tanto, desobedecer, mentir, gritar, morder y atacar pueden evolucionar en trastorno de conducta antisocial, violencia interpersonal y criminalización en la adolescencia^(26-27)^.

Los profesionales del presente estudio enfatizaron que hay que explicarle el “por qué no”, considerando que el niño se está desarrollando y necesita ese diálogo. Esta actitud educativa coincide con la presente en la literatura que recomienda el uso de respuestas cortas durante el período disciplinario del niño, ya que su desarrollo cerebral es gradual y la corteza prefrontal, responsable de las conductas de control cognitivo, sólo alcanza la madurez en la edad adulta^(28)^. Hay que darle una explicación específica y simple de la acción esperada, que diga claramente qué hacer en lugar de qué no hacer^(11,29)^.

Los profesionales destacaron la importancia de “preguntar cómo se siente el otro niño” en casos de agresión entre niños, para enseñarle empatía. La empatía es crucial para la autorregulación y el desarrollo socioemocional saludable^(25)^. Los padres deben tener estilos de crianza consistentes, ya que diferentes grados de control pueden afectar negativamente el desarrollo de la empatía y fomentar conductas antisociales^(30-31)^. Según Bronfenbrenner, en el microsistema las relaciones frecuentes y continuas son esenciales para un desarrollo saludable, y reproducen lo aprendido en el mesosistema^(6)^.

El tiempo de exposición a los medios de comunicación fue un tema de la guía que llamó la atención de los profesionales y se asocia directamente con las dificultades de autorregulación emocional durante el desarrollo del niño^(32)^. La exposición a videojuegos violentos aumenta el riesgo de desensibilización a la violencia, puede aumentar la agresividad y disminuir el comportamiento prosocial^(33)^. En este sentido, otros investigadores también han demostrado que el uso problemático de las redes sociales está directamente relacionado con bajos niveles de cercanía entre padres e hijos^(34)^. Además, en la teoría bioecológica del desarrollo humano, todos los sistemas influyen en la formación de una persona. Se moldean en el entorno en el que el niño se encuentra e interactúa, ya sea de forma directa o indirecta^(6)^.

El uso de la guía del *Programa Brincar Legal* mejoró y contribuyó a fijar contenidos, que fueron puestos en práctica en el día a día de los profesionales de los diferentes escenarios de atención infantil y familiar. Esta guía también se consideró práctica y fácil de aplicar para los padres colombianos, también redujo significativamente el uso del castigo físico y la creencia de que el castigo es la mejor alternativa para educar y controlar la conducta de los niños^(8)^.

Los profesionales destacaron dilemas relacionados con el proceso de disciplinar a los niños, ya que enseñarles a los niños cómo comportarse es un proceso continuo de orientación que se desarrolla durante meses y años y requiere mucha paciencia. La literatura afirma que la forma de educar está influenciada por las experiencias, el contexto sociohistórico-cultural, la ideología y el entorno^(6)^. Debido a la complejidad del proceso de educar a un niño, es recomendable considerar la transmisión de actitudes saludables para que no se perpetúen conductas violentas. Para ello, se puede utilizar el enfoque parental positivo para reforzar los factores protectores^(35)^.

Las dificultades observadas en el proceso de acercamiento de los profesionales a los padres/cuidadores para gestionar la discusión de la guía, pueden estar relacionadas con el hecho de que es necesario que tengan una mayor apropiación y habilidad en el tema, lo ideal sería que abordaran el tema desde que se gradúan y a lo largo de la educación continua en las instituciones, ya que la violencia contra los niños sigue siendo uno de los mayores problemas de nuestra sociedad y es urgente que se implementen diferentes acciones para romper la cadena de violencia.

La intolerancia a la violencia se manifestó en los resultados de una investigación nacional que identificó un aumento de las denuncias al número asignado para denunciar violaciones a los derechos humanos en Brasil en 2021, que registró 186.862 casos de violaciones a los derechos de los niños en el ámbito doméstico, que incluyen maltratos, exposición, lesiones corporales, tortura psicológica, amenazas, feminicidio, *bullying*, entre otros^(23)^.

La contribución que realiza el presente estudio al tema se destaca debido a la escasez de programas parentales en el país destinados a prevenir la violencia contra los niños^(23)^. Considerando la opinión de los profesionales de la educación y la salud sobre la guía de un Programa viable que pueda ser aplicado a padres/cuidadores, se cree que es posible reducir la violencia contra los niños y, por lo tanto, modificar actitudes intergeneracionales que aún toleran el castigo físico, psicológico, entre otros, para educar o disciplinar a los niños.

Además, es necesario ofrecer nuevas posibilidades para interrumpir los ciclos de violencia que se han perpetuado durante generaciones y, de esa forma, promover la cultura de paz establecida en los objetivos de desarrollo sostenible.

Se consideran limitaciones del estudio el hecho de que sea necesario utilizar el *Programa Brincar Legal* en otros escenarios, con nuevos diseños de investigación para profundidad y evaluar mejor su efectividad. Se puede tomar como ejemplo la población de padres/cuidadores en el contexto de sus hogares, lo cual era imposible en el momento que se realizó este estudio debido a las restricciones relacionadas con la pandemia de covid-19*.*

## Conclusión

Los profesionales consideraron que el *Programa Brincar Legal* era una herramienta innovadora, útil, práctica y con lenguaje simple. Aportó conocimiento, mejoró las prácticas y facilitó la difusión entre los padres/cuidadores en los entornos educativo y de salud. Se destacó porque aborda el tema de manera indirecta, evita la vergüenza y promueve una visión educativa amplia. La principal dificultad fue recordar y aplicar todo el contenido, lo que se pudo superar incorporando la guía al trabajo diario.

Contar con un programa breve e integral les permite a los profesionales tener mayor seguridad en su trabajo y ayuda a las instituciones en las que trabajan a ser más efectivas en la protección de los niños, por ejemplo, en la atención primaria, durante las consultas de puericultura y en la atención para la salud del niño en edad escolar, para prevenir la violencia.

Por lo tanto, contribuye a la población general y al niño, ya que las prácticas parentales saludables pueden influir positivamente en el desarrollo del sistema bioecológico. Por ende, el Programa también fortalece el desarrollo sostenible, protegiendo a los niños de la violencia, promoviendo la paz y las instituciones efectivas.

# Translation: Programa Brincar Legal na prevenção da violência contra crianças: fortalecendo o desenvolvimento sustentável^*^

**DOI:** 10.1590/1518-8345.7320.4435

**Authors:** Janaina Recanello Begui, Andressa Larissa Dias Müller de Souza, Naiara Barros Polita, Maria de Fátima Garcia Lopes Merino, Adriana Valongo Zani, Rosângela Aparecida Pimenta

**Affiliations:** 1 Universidade Estadual do Norte do Paraná, Departamento de Enfermagem, Bandeirantes, PR, Brasil.; 2 Universidade Estadual de Londrina, Londrina, PR, Brasil.; 3 Universidade Estadual de Londrina, Departamento de Enfermagem, Londrina, PR, Brasil.; 4 Universidade Estadual de Maringá, Departamento de Enfermagem, Maringá, PR, Brasil.

**Keywords:** Proteção da Criança, Maus Tratos Infantis, Responsabilidade Parental, Educação Infantil, Atenção Primária à Saúde, Desenvolvimento Sustentável

## Abstract

**Objetivo:**

compreender a percepção de professores e profissionais de saúde sobre o uso do Programa Brincar Legal para pais/cuidadores para a prevenção da violência contra crianças.

**Método:**

estudo qualitativo, descritivo e exploratório realizado por meio de três grupos focais com vinte professores da educação básica e profissionais da atenção primária à saúde que aplicaram o Programa para pais/cuidadores, em 2022. A análise de discurso de linha francesa orientou a análise de dados à luz da teoria de Urie Bronfenbrenner.

**Resultados:**

a partir da percepção dos participantes, emergiram três categorias: inovação e utilidade: aplicabilidade da teoria na prática; ressignificando atitudes de austeridade e impaciência contra a criança; disciplinar a criança e não punir: dilemas e desafios. Os depoimentos evidenciaram que o Programa é inovador, de acesso rápido, linguagem clara e de fácil aplicabilidade na prática para pais/cuidadores.

**Conclusão:**

o uso do Programa agregou conhecimento e propiciou atitudes positivas para o desenvolvimento do sistema bioecológico quanto ao enfrentamento e prevenção da violência contra a criança.

## Destaques

**(1)** Fortalece o desenvolvimento sustentável, protegendo a criança da violência.

**(2)** O programa promove a paz e instituições mais eficazes na proteção infantil.

**(3)** Percepção do Programa: inovador, aplicável à prática, breve e de fácil linguagem.

**(4)** Ferramenta relevante para profissionais que atuam na atenção à criança e sua família.

**(5)** Fomenta ações assertivas para prevenção da violência contra crianças.

## Introdução

A violência contra a criança impacta negativamente seu desenvolvimento físico, cognitivo e socioemocional, trazendo consequências graves desde a infância até a idade adulta^(1)^. Sua prevenção está listada como uma das metas dos Objetivos de Desenvolvimento Sustentável (ODS), que, para o Brasil, prevê “proteger todas as crianças e adolescentes do abuso, exploração, tráfico, tortura e todas as outras formas de violência”^(2)^.

O número de casos notificados no mundo não reflete a realidade devido à subnotificação, ocultando essa crise global, possivelmente ligada à fragilidade da rede de proteção^(3)^. Situação que agravou durante a pandemia de covid-19, ao expor crianças e adolescentes a mais violência doméstica, por serem obrigados a conviver por mais tempo em casa e afastados de ambientes protetores, como as escolas^(4-5)^.

Segundo a teoria bioecológica de Urie Bronfenbrenner, a linguagem, os símbolos, a interação pessoa-pessoa e pessoa-contexto, ao longo do tempo (cronossistema), também os aspectos biológicos, cognitivos, emocionais e comportamentais, influenciam o desenvolvimento humano e familiar. O processo de interação ocorre em contextos distintos presentes em sistemas que são dinâmicos e se inter-relacionam, sendo eles o microssistema, mesossistema, exossistema e macrossistema. Portanto, o processo, pessoa, contexto e tempo explicam o dinamismo sistêmico da teoria bioecológica, que pode ser utilizada para compreender que a criança que sofre violência terá alterações no seu desenvolvimento, percepção de mundo e comportamento^(6)^. Como, por exemplo, vítimas de violência na infância frequentemente manifestam comportamentos agressivos, aumentando o risco de perpetração desse comportamento^(7)^.

Portanto, urge capacitar os envolvidos na proteção dos direitos das crianças para prevenir, identificar e intervir precocemente^(3)^. Programas parentais podem trazer benefícios a curto e longo prazo^(8)^, configuram-se uma estratégia na prevenção de maus-tratos, embora sejam escassos no Brasil em comparação com países desenvolvidos como Canadá e Estados Unidos (EUA)^(9-10)^. No Tennessee-EUA, no *Vanderbilt University Medical Center*, foi desenvolvido o *Play Nicely Program: The Healthy Discipline Handbook* pelo pediatra Seth Scholer, um programa universal de intervenção breve que apresenta práticas parentais para mitigação da violência física contra a criança menor de onze anos, composto por um guia e uma multimídia interativa educacional^(11)^.

O guia do *Play Nicely Program: The Healthy Discipline Handbook,* traduzido e adaptado transculturalmente para o Brasil, mediante etapas metodológicas de traduções independentes, retrotradução, avaliação por especialistas e pré-teste, passou a ser denominado “Programa Brincar Legal: o guia de disciplina saudável”^(12)^. Ele permanece vinculado à Vanderbilt University, estando disponível gratuitamente para fins educacionais e de pesquisa^(13)^.

O Programa apresenta cinco passos para realizar ante atos de agressão contra a criança e problemas comportamentais, como a reprodução de atos/símbolos de violência física contra seus colegas na escola, sendo eles: I-ensine seu filho a não ser uma vítima; II-aprenda formas de agir frente aos comportamentos/desafiadores; III-diminua a exposição à violência e à mídia em excesso; IV-demonstre amor; V-seja firme^(11)^. Ensina práticas disciplinares saudáveis para pais, professores e profissionais de saúde, exemplificando 20 opções para lidarem com crianças, indicando quais ações são boas opções e quais não são recomendadas, visando que não haja atitudes punitivas gerando outro tipo de violência como a psicológica, física ou ambas^(11)^.

Estudos que avaliaram pré-intervenção e pós-intervenção utilizando o Programa identificaram diminuição no uso do castigo físico contra a criança, ocasionado pelos pais^(8,14-16)^. Também, pais afirmaram que, após aprenderem com o Programa, modificaram suas atitudes de disciplina, para mais conversa/explicação, mais redirecionamento, mais paciência/escuta e menos raiva/gritos^(15-16)^. Um estudo que avaliou pré e pós-treinamento usando o Programa para capacitar profissionais de saúde identificou uma melhora significativa na capacidade de aconselhar os pais a respeito de estratégias disciplinares apropriadas, desencorajando o uso de agressão física^(17)^. Essas pesquisas foram realizadas em outros países, visto que o presente estudo é o primeiro a utilizar o Programa no Brasil.

A violência contra a criança caracteriza-se pela sua ocorrência na infância, seja ela ocasionada por adultos, seja por outras crianças, reconhecidamente classificadas nos tipos física, psicológica, sexual, negligência e institucional^(18)^. Considerando essa problemática mundial e a inexistência neste país de programas gratuitos de intervenção breve para prevenção da violência física contra a criança, este estudo contribui apresentando uma ferramenta para orientar práticas disciplinares saudáveis aos pais, colaborando na prevenção da violência. O Programa pode ser uma alternativa para auxiliar as políticas brasileiras de proteção da criança e fortalecer o cumprimento dos ODS nos espaços de cuidado educacional e saúde que atendem crianças e suas famílias. Neste sentido, este estudo objetiva compreender a percepção de professores e profissionais de saúde sobre o uso do Programa Brincar Legal para pais/cuidadores para a prevenção da violência contra crianças.

## Método

### Delineamento do estudo

Trata-se de um estudo qualitativo, descritivo e exploratório, que atendeu às recomendações dos Critérios Consolidados de Relato de Pesquisa Qualitativa (COREQ).

### Cenário

Estudo realizado em um município no norte do Estado do Paraná, Brasil, em três instituições: Centro Municipal de Educação Infantil (CEMEI), que atende crianças na faixa etária de seis meses a quatro anos; Unidade Básica de Saúde (UBS) e; o Centro de Referência da Assistência Social (CRAS), que atende famílias em situação de vulnerabilidade social e inseridas em um Programa do governo federal intitulado “Criança Feliz”, cujo objetivo é acompanhar e fortalecer o desenvolvimento integral das crianças na primeira infância, estimulando o vínculo familiar e comunitário^(19)^.

### Período

A pesquisa foi realizada nos meses de fevereiro a abril de 2022.

### População

Profissionais de saúde e de educação.

### Critérios de seleção

Os critérios de inclusão para os profissionais da educação foram ser professor(a) da educação básica e/ou pedagogo(a), atuando com crianças na faixa etária de um a dez anos. Para os profissionais de saúde, os critérios foram atuar diretamente com famílias compostas por pelo menos um filho na faixa etária referida anteriormente, sendo os pais/cuidadores alfabetizados e não terem deficiência visual ou cognitiva. Todos os profissionais precisariam participar das duas etapas da pesquisa. Os critérios de exclusão para os profissionais foram estar usufruindo de férias ou licenças.

### Participantes

A amostragem foi por conveniência e o recrutamento foi realizado pela pesquisadora principal, que conversou presencialmente com a coordenação de cada equipe. Posteriormente, a coordenadora do CMEI, por meio do aplicativo *WhatsApp*, convidou os dez professores da instituição. Na UBS, a coordenação sugeriu abordar diretamente os profissionais, e nove foram convidados individualmente. No CRAS, a coordenadora fez uma reunião com os seis profissionais e explicou sobre o estudo. Todos os profissionais aceitaram participar, mas no segundo encontro houve perda de cinco pessoas: no CEMEI, um professor por rescisão contratual, e na UBS participantes desistiram por não aplicarem o guia do Programa.

### Coleta de dados

A coleta de dados foi conduzida apenas pela pesquisadora principal, que durante o estudo tinha titulação de mestre, atuava como docente na graduação em enfermagem em universidade pública, com experiência na técnica de grupo focal, não possuía nenhum vínculo com as instituições, nem conhecia os participantes previamente à pesquisa. Utilizaram-se distanciamento, máscara e álcool gel, recomendados para evitar a disseminação e contaminação da covid-19^(20)^.

A coleta de dados foi realizada em dois momentos com os mesmos participantes, em cada local de trabalho (CMEI, UBS, CRAS), por meio de grupo focal. Os encontros ocorreram em salas que possuíam mesas retangulares com cadeiras, janelas e poucos ruídos externos. A privacidade se deu pelo uso de portas fechadas e ambiente arejado, estando presentes apenas os participantes e a pesquisadora.

Não foi realizado teste-piloto. No primeiro encontro ocorreram: a explanação do objetivo do estudo; explicação do termo de consentimento livre e esclarecido; coleta das assinaturas; preenchimento individual de um questionário sociodemográfico; realização do grupo focal para conhecer a percepção dos profissionais acerca da violência contra a criança; explicação e a entrega do material para uso do Programa na rotina diária. Neste primeiro momento, o grupo focal teve maior duração, visto que foram necessários 30 minutos para a explanação sobre o programa.

Nesse primeiro momento, o grupo participou ativamente da discussão e o tempo foi mensurado por um cronômetro, sendo 47 minutos no CMEI, 67 minutos na UBS e 59 minutos no CRAS. Os participantes foram orientados a utilizar o Programa por 35 dias, mas não havia um número mínimo de atendimentos determinado. Foi solicitado para os professores aplicarem em sala de aula com as crianças no CMEI e para os profissionais do CRAS e da UBS orientarem os pais/cuidadores sobre as estratégias de disciplina saudável para lidar com as atitudes de desobediência e indisciplina de seus filhos.

O segundo momento aconteceu após o término do período de uso do material. Realizou-se um grupo focal em cada local utilizando um roteiro semiestruturado, contendo as seguintes perguntas: o que significou para você utilizar o guia Brincar Legal? Você já tinha ouvido sobre disciplinas saudáveis? Se sim, conte-me mais sobre isso. Quais foram as contribuições que o guia trouxe para sua prática profissional? Qual sua percepção em relação ao seu conhecimento prévio e ao conhecimento adquirido após a utilização do guia? Houve dificuldades na aplicação do material? Se sim, quais foram?

O grupo focal teve duração de 28 minutos no CMEI, 30 minutos na UBS e 25 minutos no CRAS. Nessa etapa, houve perda de cinco participantes, mas os demais colaboraram ativamente na discussão.

Em ambos os momentos de grupo focal, as entrevistas coletivas foram gravadas por dois aparelhos de áudio, transcritas na íntegra por uma equipe especializada e não foi utilizado o diário de campo. As transcrições foram disponibilizadas aos participantes, mas nenhum quis lê-las para tecer comentários ou correção. Os resultados não foram apresentados aos participantes.

### Tratamento e análise dos dados

Os dados foram analisados e explorados a partir da análise de discurso de Michel Pêcheux sob o olhar de Eni Orlandi^(21)^, sem a utilização de *software,* e discutidos à luz da teoria bioecológica do desenvolvimento humano de Urie Bronfenbrenner^(6)^. Esse tipo de análise foi escolhido por considerar o que não está transparente, sendo o sujeito dotado de ideologias e marcas históricas, de modo que o real é afetado pelo simbólico e a pessoa não tem o controle e a consciência do quanto sua linguagem e história são afetadas.

Foi realizada a dessuperficialização do *corpus*, ou seja, do material bruto (superfície linguística) resultante da transcrição. Esta etapa consiste na análise linguística do material, como, por exemplo, quem diz, como diz e em que circunstâncias ele diz, e durante ela foram realizadas as marcações textuais de sinonímias, gerando 103 codificadores. A partir deles, ocorreu a passagem da superfície linguística para o objeto discursivo, o qual é afetado por memórias e experiências que estão implícitas no discurso. A terceira etapa é a passagem do objeto discursivo para o processo discursivo, ou seja, o movimento de compreensão e de formação ideológica, na qual emergiram as categorias ^(Figura 1)^.

**Figura 1:** - Fluxograma do dispositivo analítico, adaptado de Orlandi^(21)^

### Aspectos éticos

O estudo atendeu às normas de ética e foi aprovado pelo Comitê de Ética em Pesquisa com parecer número 3.970.750. Visando não expor os participantes, eles foram identificados pela letra P e um número, foram feitas correções ortográficas e retirados os vícios de linguagem, sem alteração do fluxo.

## Resultados

Dos 20 participantes, a totalidade foi do sexo feminino, a idade variou entre 23 e 56 anos, a maioria era casada, com filhos em idade de um a 40 anos e com ensino superior completo, sendo, nove professoras, três assistentes sociais e três pedagogas, duas enfermeiras, uma agente comunitária de saúde, uma técnica de enfermagem e uma psicóloga. O tempo de profissão variou de três meses a 20 anos.

Com base na concepção de que a linguagem não é transparente e que todo discurso é interpelado pela história e pela ideologia do sujeito, buscou-se, no processo de análise, a compreensão da língua fazendo sentido. Dessa forma, o processo discursivo mostrou, por meio do conhecimento intrínseco e extrínseco de cada pessoa, a percepção de que o uso do guia do Programa representou para eles inovação, utilidade, aplicabilidade, formas de agir diante de comportamentos desafiadores, dilemas e desafios.

### Categoria I – Inovação e Utilidade: aplicabilidade da teoria na prática

O guia se apresentou como uma nova ferramenta de aporte científico e inovadora no ambiente de trabalho. Tem potencial de proporcionar melhorias práticas e somar às que já existem para prevenção ou combate à violência física contra a criança:

[...] *você tem mais coisa, mais material para elaborar atividade, para elaborar orientação para os pais, é um material que veio a somar no nosso trabalho* (P1); *abre um leque de opções, a gente não fica numa opção só, agora a gente tem mais opções para procurar mais conhecimento* (P18).

Para que algo se torne útil, é necessário satisfazer uma realidade local, que apresente vantagens e seja proveitoso. Os participantes perceberam o uso do material em sua realidade diária, desde a sua abrangência e prioridades, conteúdos, linguagens e orientações:

[...] *abordagens simples de entender, fácil, uma linguagem fácil que a gente pode estar passando para os pais de uma maneira que eles entendam e que não ofenda* (P3).

O guia foi destacado como um conjunto de conhecimentos, de procedimentos que priorizam orientações aos pais/cuidadores em suas ações cotidianas e relacionadas ao processo disciplinar. Além disso, apresentou um conteúdo útil na elaboração de atividade indireta, ou seja, na impossibilidade de um contato direto com os cuidadores, como em casos de pandemia, ao poder ser utilizado para atendimento em ambientes virtuais.

*Foi feito um panfleto de orientação, de forma abrangente, para todos, e foi entregue para cada família, e então, depois nos atendimentos remotos, vamos fazendo o* feedback (P1).

A utilização do guia possibilitou novos caminhos para orientações desafiadoras no dia a dia em sala de aula:

[...] *quando a criança morde, bate na outra, foi muito útil aqui a gente saber o que, como a gente deve lidar* (P8); *Eu utilizei, porque as minhas crianças têm dois aninhos, eles batem no amiguinho, ficam mordendo, então é, agora eu sei como chegar, conversar, agir* (P14).

Tido como um material que veio para somar no trabalho diário, o guia apresentou outros desdobramentos, como exemplo a possibilidade de novas ideias na abordagem de outros tipos de violência, por meio de vídeos, músicas, confecção e orientação de panfletos:

*Tiramos até ideias…a gente não tinha essa ideia de mandar atividade assim, mas depois do guia! Tem um vídeo que mostra, uma musiquinha, mostrando quem pode relar no corpo da criança, então a gente nunca tinha pensado nisso, depois do livrinho a gente está procurando* (P4).

Uma das atividades do Programa é também abordar pessoas que foram pais/cuidadores de crianças sobre a violência e permitiu dialogar e entender a maneira de educar das gerações passadas:

*Eu achei interessante, eu falei com uma avó e eu até achei que ela fosse responder de bater também, porque ela teve essa educação, mas não...dela não querer passar para menina, a maneira que ela foi criada* (P19).

Quanto ao processo de aprender e apreender algo, o guia do Programa foi considerado de fácil aplicação, propiciou um novo olhar para implementar ações no processo de educar, uma fonte de conhecimentos específica e com base científica de como e quando realizar:

*O conhecimento que eu tinha, era da teoria. A gente não sabe como colocar aquilo ali na prática a hora que você tiver diante de alguma situação. O guia traz, de forma mais prática, sugestões de como fazer. Dá exemplos das situações que a gente pode estar vendo e como conduzir. Eu acho que essa é a grande diferença desse material* (P1).

Durante o uso do guia do Programa pelos profissionais, uma das ações foi a de trabalhar diretamente com as famílias de maneira gentil e sem constranger os pais/cuidador, o que auxiliou em como falar, sugerir e orientar. Além disso, o acesso imediato ao material impresso para consulta sempre que necessário facilitou a utilização cotidiana.

Alguns profissionais de saúde abordaram pais/cuidadores sobre “bater em seu filho” durante o atendimento, surpreendidos com as respostas de que não puniam seus filhos para educá-los:

*Eu me surpreendi também com as respostas das mães que eu entrevistei. A gente sempre tem aquela mentalidade de que a maioria das pessoas é conivente com bater e gritar para educar a criança* (P8).

Por outro lado, o profissional de saúde pode vivenciar a contradição na resposta da mãe em detrimento da criança, quando ela afirmou não punir e seu filho referiu que era punido fisicamente por ela:

*Eu perguntei para a mãe e a criança estava perto, e aí a mãe falava que não batia, e a criança falava “não, a mãe bate sim”. Então aquela controvérsia, quer dizer, a criança realmente está falando a verdade. Aí eu perguntei para ela, ela falou assim: “ah, de vez em quando a gente tem que bater, não tem como, não tem”* (P18).

### Categoria II - Ressignificando atitudes de austeridade e impaciência contra a criança

Quanto ao conteúdo, destacou-se que, por orientar os pais/cuidadores em como proceder nos casos de agressão de seus filhos por meio de uma abordagem legal, bem como o fato de considerar a criança como um ser humano integral e em desenvolvimento, o guia também favorece a empatia e humanização:

*O guia traz pontualmente como falar diante de tal situação, a forma de conduzir, então é mais fácil, ele dá exemplos das situações que podemos encontrar e como proceder* (P1).

Os profissionais também destacaram alguns temas e opções de orientações que o guia oferece, tais como a desobediência e a mentira:

*O que escuto bastante é a mãe reclamando da criança desobediente. Então, talvez no ponto que fala da desobediência, da criança mentir,* dê *para a gente trabalhar mais coisas nesse sentido* (P5).

O entendimento do “não” como uso frequente para interrupção dos momentos de desobediência da criança e o estabelecimento de regras claras, curtas e firmes também foi mencionado:

[...] *aquela disciplina do não, não basta falar “não” para a criança, você tem que explicar o porquê do não. Achei bem interessante. É uma coisa simples que faz a diferença* (P8).

O guia mostrou o caminho e deu maior direcionamento de como lidar com as situações, além de despertar um olhar mais atento aos pais/cuidadores no momento do atendimento, facilitando o diálogo com o cuidador da criança. A estratégia de redirecionar o comportamento está representada na fala a seguir:

*Tem uma parte que eu achei interessante, é de como usar o nosso corpo, por exemplo a mão, a criança foi lá e bateu, não, vamos usar a mãozinha para fazer outra coisa. A*í *chutou um amigo, não pera lá, o seu pé é feito para chutar a bola, vamos chutar a bola, jogar um futebol, pular uma amarelinha…* (P4).

Os entrevistados trouxeram a estratégia de preocupar-se com os sentimentos dos outros, proporcionando maior empatia:

[…] *ela colocou como primeira opção “perguntar ao seu filho como a outra criança se sente”, a única que respondeu, a única que teoricamente passaria por parte da humanização, da empatia, do ser humano* (P16).

Alguns conteúdos destacados do guia remetem à demonstração de amor e de assegurar um tempo de qualidade com os filhos. Nesse sentido, foi abordada a escassez da presença dos pais/cuidadores, muitas vezes relacionada ao tempo despendido no trabalho, gerando estresse e comprometimento das relações.

Foi ressaltada a visibilidade que o guia proporcionou acerca da importância dos cuidadores terem uma mesma linguagem com a criança e saberem abordar sem assustá-la, pois muitos pais/cuidadores “gritam” no ato disciplinar, podendo resultar no insucesso da disciplina, bem como afetar a sua segurança emocional.

O controle do tempo diário das mídias e as práticas esportivas no processo educativo da criança também foi destacado pelos profissionais:

*Achei legal da televisão, do* tablet*, que precisamos colocar um limite, a gente sabe que é melhor, e o guia fala sobre práticas esportivas também como opção. Bem legal, achei interessante* (P15).

### Categoria III – Disciplinar a criança e não punir: dilemas e desafios

Os entrevistados também trouxeram os dilemas vivenciados no processo educativo, as dificuldades em estabelecer outras regras de disciplina, como deixar no quarto, tirar o celular, ou seja, como utilizar outros meios de educar:

*A mãe diz que conversa bastante, mas sempre pune. Não bate, mas coloca de castigo e faz a criança refletir. Bater ela é contra, geralmente deixa no quarto, tira celular, enfim, alguma coisa faz* (P19).

O “bater em determinadas situações” ainda foi citado como um dilema, situações que dependem do senso de justiça e injustiça, defesa e ataque, que se relacionam com valores pessoais e familiares:

[...] *se a outra criança bater na minha filha, eu bateria na minha filha, porque alguma coisa ela fez, então vou educar ela, batendo nela* (P20).

Houve ainda dúvidas em relação à questão de revidar ou não quando acontece uma agressão:

*Uma mãe falou para mim que ela incentiva a filha dela a revidar, porque o filho dela mais velho, de 15 anos, foi “saco de pancada” na escola e ela não quer que aconteça a mesma coisa com a menina dela, que tem 8 anos hoje, então ela ensina que qualquer coisa que fizer é para a menina fazer a mesma coisa* (P19).

Outro dilema no processo da educação parental foi como ter paciência, pois a educação é um processo longo e repetitivo, já que a criança não entende tudo na primeira vez:

*Eu acho que a dificuldade é sempre estar ali batendo na mesma tecla, porque às vezes é uma palavra, igual com meu filho que não é acostumado a ouvir, daí você tem que estar sempre batendo na mesma tecla, até eles entenderem* (P9).

Em relação aos desafios, os profissionais relataram dificuldade durante o atendimento para abordar o tema violência antes de explicar o guia e também em interferir quando se iniciava uma discussão sobre práticas disciplinares não recomendadas.

Para alguns profissionais que trabalham diretamente com as famílias participantes de um programa social de maneira voluntária, houve receio em utilizar o guia em sua totalidade de maneira inicial por temerem sua saída. Trata-se de um tema delicado e, dependendo da abordagem, os pais/cuidadores podem interpretar como uma atitude invasiva na forma como educam e punem seus filhos:

*A gente não tem como interferir muito, sabe? Esse informativo (panfleto) foi feito de forma abrangente, para todos, como orientação. Se a família percebe que a gente está querendo palpitar ou falar que ela está fazendo errado, não gostam e falam: “oh,* não quero mais você aqui” (P1).

Em contrapartida, alguns entrevistados trouxeram também as dificuldades em lembrar tudo na hora de aplicar e não conseguir aplicar na prática o que aprenderam:

*Não dá para gente lembrar tudo, mas um pouco dá para gente continuar aplicando, o livro a gente tem que ler, reler e ler de novo, a gente sempre vai consultando* (P9).

Mesmo diante da pontuação destes dilemas, afirmaram gostar do guia, considerando-o “legal” e “interessante”, válido para orientar os pais/cuidadores. Houve também uma boa aceitação dos pais/cuidadores participantes durante o uso do guia pelos profissionais, e demonstraram muito interesse pelo material.

## Discussão

Os programas que auxiliam os pais a desempenharem o papel parental são centrais para cumprir os objetivos dos serviços de proteção à criança, sendo recomendados como estratégias relevantes para prevenir o abuso infantil^(10,22)^. Também estimulam práticas parentais saudáveis, colaboram para a mitigação da violência e fortalecem o objetivo 16 dos ODS, que visa à promoção da paz, justiça e instituições eficazes^(2)^.

No Brasil, crianças em situação de desigualdade social enfrentam riscos de violência devido a fatores como baixo *status* socioeconômico, privação de educação, alimentação e saúde^(23)^. Nesse estudo, o Programa mostrou-se inovador no cotidiano de trabalho e a linguagem fácil favoreceu o seu uso com famílias vulneráveis atendidas no CRAS. Um ensaio clínico em Nashville (EUA) também evidenciou resultados semelhantes com famílias de baixa renda^(15)^.

Houve aprendizado sobre os tipos de violência e como abordá-los, redirecionando comportamentos agressivos para positivos, como usar o pé para chutar uma bola em vez de um colega. Atos como morder, empurrar, puxar cabelo e bater são relacionados à imaturidade e devem ser moldados para a regulação emocional da criança^(24)^. Os pais, como moduladores do comportamento, devem auxiliar a autorregulação da criança^(25)^. Ensiná-las amavelmente sobre as suas atitudes aumenta as chances de sucesso educativo^(11)^, interferindo diretamente na relação do microssistema^(6)^, promovendo uma mudança que pode resultar em atos saudáveis. Pois, desobedecer, mentir, gritar, morder e agredir podem evoluir para um transtorno de comportamento antissocial, violência interpessoal e a criminalização na adolescência^(26-27)^.

Os profissionais do presente estudo destacaram sobre explicar o “por que não”, entendendo que a criança está em desenvolvimento e precisa desse diálogo. Essa atitude educadora alinha-se à literatura, que recomenda o uso de respostas curtas durante o momento disciplinar da criança, visto que o seu desenvolvimento cerebral é gradativo e o córtex pré-frontal, responsável por comportamentos de controle cognitivo, atinge a maturidade somente na vida adulta^(28)^. Deve-se fornecer uma declaração específica e simples da ação esperada, dizendo claramente o que fazer, em vez do que não deve fazer^(11,29)^.

Os profissionais destacaram a importância de “perguntar como a outra criança se sente” em casos de agressão entre crianças, ensinando empatia. A empatia é crucial para a autorregulação e o desenvolvimento socioemocional saudável^(25)^. Pais devem ter estilos parentais consistentes, pois diferentes graus de controle podem impactar negativamente o desenvolvimento da empatia e favorecer comportamentos antissociais^(30-31)^. Segundo Bronfenbrenner, no microssistema, as relações frequentes e contínuas são essenciais para um desenvolvimento saudável, reproduzindo o apreendido no mesossistema^(6)^.

O tempo de exposição às mídias foi um tema do guia que chamou a atenção dos profissionais e está diretamente associado às dificuldades de autorregulação emocional durante o desenvolvimento da criança^(32)^. A exposição a videogames violentos eleva o risco de dessensibilização à violência, pode aumentar a agressividade e diminuir o comportamento pró-social^(33)^. Neste sentido, outros pesquisadores evidenciaram também que o uso problemático de mídias sociais está diretamente relacionado com os baixos níveis de proximidade entre pais e filhos^(34)^. Além disso, na teoria bioecológica do desenvolvimento humano, todos os sistemas influenciam a formação de uma pessoa. Eles são moldados no ambiente onde a criança se encontra e se relaciona, seja direta ou indiretamente^(6)^.

O uso do guia do Programa Brincar Legal aprimorou e contribuiu para fixar conteúdos, colocados em prática no cotidiano dos profissionais nos diferentes cenários de cuidado da criança e família. Este guia também foi considerado prático e de fácil aplicabilidade para pais colombianos, bem como houve redução significativa no uso de castigo físico e diminuição na crença de que o castigo é a melhor alternativa para educar e controlar o comportamento das crianças^(8)^.

Os dilemas foram destacados pelos profissionais no que se refere ao processo de disciplinarização de crianças, pois ensinar a elas como se comportar é um contínuo de orientação que acontece durante meses e anos, exigindo muita paciência. Nesse contexto, evidencia-se na literatura que a maneira de educar é influenciada pelas experiências, contexto sócio-histórico-cultural, ideologia e ambiente^(6)^. Em meio à complexidade do processo de educar uma criança, é prudente considerar a transmissão de atitudes saudáveis para que o comportamento violento não se perpetue. Para tanto, a abordagem parental positiva pode ser utilizada para o fortalecimento de fatores de proteção^(35)^.

Em relação às dificuldades encontradas no processo de abordagem dos pais/cuidadores pelos profissionais para manejar a discussão do guia, elas podem estar relacionadas com a necessidade de maior apropriação e habilidade com o tema. Idealmente, desde o período da graduação até a educação permanente nas instituições em que atuam, a violência contra a criança ainda é um dos maiores agravos na nossa sociedade e urge implementar diferentes ações para romper a cadeia de violência.

A intolerância à violência manifestou-se nos resultados de pesquisa nacional que identificou aumento de denúncias no Disque 100, no ano de 2021, registrando 186.862 casos de violações de direitos de crianças no ambiente doméstico, sendo elas maus tratos, exposição, lesão corporal, tortura psíquica, ameaça, feminicídio, *bullying*, entre outras^(23)^.

Em face da escassez de programas parentais no país voltados para a prevenção da violência contra a criança^(23)^, destaca-se a contribuição do presente estudo. Considerando a voz de profissionais de educação e saúde sobre o guia de um programa factível para ser aplicado aos pais/cuidadores, vislumbra-se mitigar a violência contra seus filhos e, por conseguinte, ajustar atitudes intergeracionais que ainda toleram a punição física, psicológica, entre outras, para educar ou disciplinar uma criança.

Além disso, é preciso oferecer novas possibilidades para haver interrupção dos ciclos de violência que têm se perpetuado ao longo das gerações e, assim, promover a cultura da paz como estabelecido nos objetivos do desenvolvimento sustentável.

As limitações do estudo referem-se à necessidade do uso do Programa Brincar Legal em outros cenários, com novos delineamentos de pesquisa para maior aprofundamento e avaliação da sua efetividade. Pode-se tomar como exemplo a população de pais/cuidadores no contexto dos seus lares, impossibilitada no momento deste estudo devido às restrições referentes à pandemia de covid-19*.*

## Conclusão

O Programa Brincar Legal foi visto pelos profissionais como uma ferramenta inovadora, útil, prática e com fácil linguagem. Agregou conhecimento, aprimorou práticas e facilitou a disseminação entre pais/cuidadores nos cenários de educação e saúde. Destacou-se por abordar indiretamente o tema, evitando constrangimento e promovendo uma visão educacional ampla. A principal dificuldade foi lembrar e aplicar todo o conteúdo, superável com a incorporação do guia no cotidiano.

A obtenção de um programa breve e abrangente possibilita aos profissionais obter maior segurança em suas ações, colaborando para que as instituições em que atuam sejam mais efetivas na proteção infantil, por exemplo, na atenção primária, durante as consultas de puericultura e no cuidado à saúde do escolar, visando a prevenção da violência.

Por conseguinte, o Programa contribui para a população geral e a criança, pois as práticas parentais saudáveis podem exercer influência positiva no desenvolvimento do sistema bioecológico. Sendo assim, ele também fortalece o desenvolvimento sustentável, protegendo a criança da violência, promovendo paz e instituições eficazes.
